# Supplementary material for: Green Synthesis of FexOy Nanoparticles with Potential Antioxidant Properties
Source: Nanomaterials (Basel). 2022 Jul 17;12(14):2449. doi: 10.3390/nano12142449 (PMC9315626; doi:10.3390/nano12142449)
Supplement: Supplementary file 1 [file nanomaterials-12-02449-s001.zip › nanomaterials-1802029-supplementary.pdf]

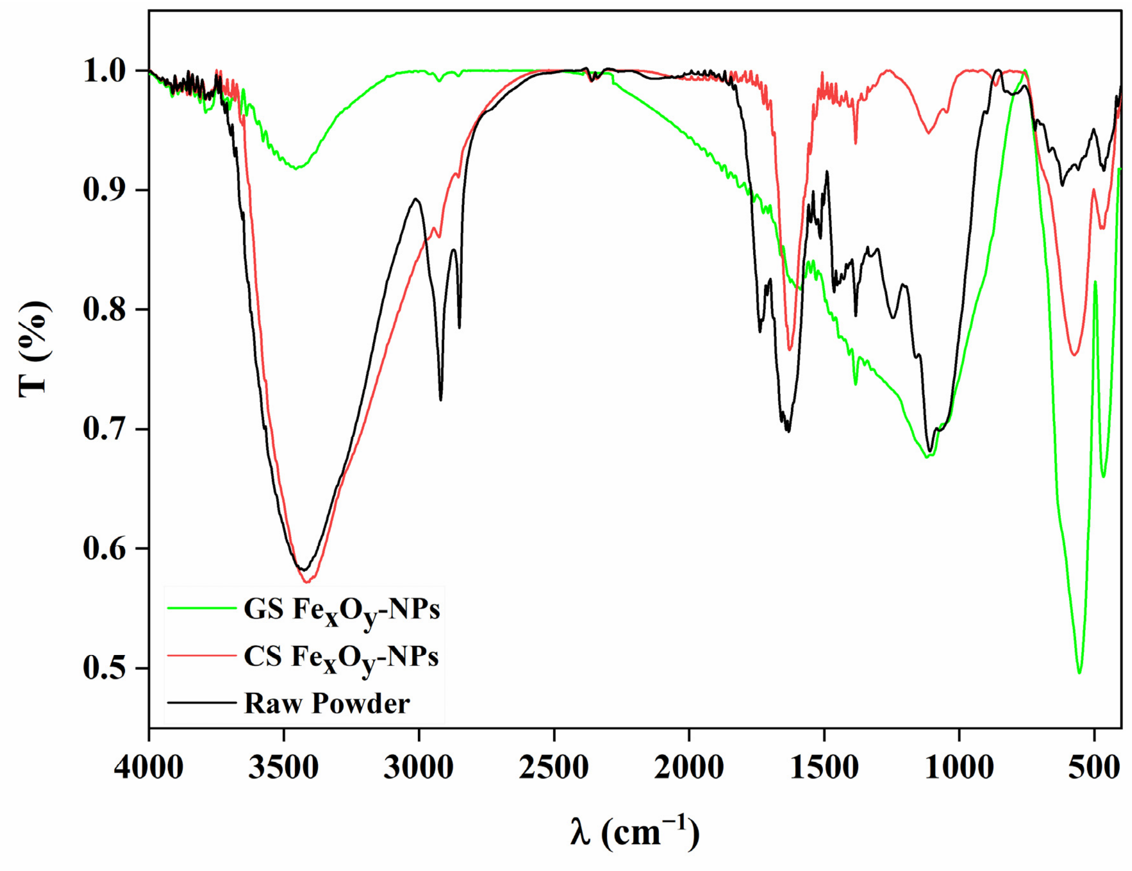

Figure S1: FTIR of the GS Fe_xO_y-NPs compared with its raw material powder and CS-Fe_xO_y-NPs.
